# Platform for ergonomic intraoral photodynamic therapy using low-cost, modular 3D-printed components: Design, comfort and clinical evaluation

**DOI:** 10.1038/s41598-019-51859-6

**Published:** 2019-11-01

**Authors:** Srivalleesha Mallidi, Amjad P. Khan, Hui Liu, Liam Daly, Grant Rudd, Paola Leon, Shakir Khan, Bilal M. A. Hussain, Syed A. Hasan, Shahid A. Siddique, Kafil Akhtar, Meredith August, Maria Troulis, Filip Cuckov, Jonathan P. Celli, Tayyaba Hasan

**Affiliations:** 1 Wellman Center for Photomedicine, Massachusetts General Hospital, Harvard Medical School, Boston, Massachusetts USA; 2 Department of Physics, University of Massachusetts at Boston, Boston, Massachusetts USA; 3 Department of Engineering, University of Massachusetts at Boston, Boston, Massachusetts USA; 4 Department of Radiotherapy, Jawaharlal Nehru Medical College, Aligarh Muslim University, Aligarh, India; 5 Department of Oto-Rhino-Laryngology, Jawaharlal Nehru Medical College, Aligarh Muslim University, Aligarh, India; 6 Department of Pathology, Jawaharlal Nehru Medical College, Aligarh Muslim University, Aligarh, India; 7 Department of Oral and Maxillofacial Surgery, Massachusetts General Hospital, Boston, MA USA; 8 Present Address: Department of Biomedical Engineering, Tufts University Medford Massachusetts, Massachusetts, USA

**Keywords:** Oral cancer, Lasers, LEDs and light sources

## Abstract

Oral cancer prevalence is increasing at an alarming rate worldwide, especially in developing countries which lack the medical infrastructure to manage it. For example, the oral cancer burden in India has been identified as a public health crisis. The high expense and logistical barriers to obtaining treatment with surgery, radiotherapy and chemotherapy often result in progression to unmanageable late stage disease with high morbidity. Even when curative, these approaches can be cosmetically and functionally disfiguring with extensive side effects. An alternate effective therapy for oral cancer is a light based spatially-targeted cytotoxic therapy called photodynamic therapy (PDT). Despite excellent healing of the oral mucosa in PDT, a lack of robust enabling technology for intraoral light delivery has limited its broader implementation. Leveraging advances in 3D printing, we have developed an intraoral light delivery system consisting of modular 3D printed light applicators with pre-calibrated dosimetry and mouth props that can be utilized to perform PDT in conscious subjects without the need of extensive infrastructure or manual positioning of an optical fiber. To evaluate the stability of the light applicators, we utilized an endoscope in lieu of the optical fiber to monitor motion in the fiducial markers. Here we showcase the stability (less than 2 mm deviation in both horizontal and vertical axis) and ergonomics of our applicators in delivering light precisely to the target location in ten healthy volunteers. We also demonstrate in five subjects with T1N0M0 oral lesions that our applicators coupled with a low-cost fiber coupled LED-based light source served as a complete platform for intraoral light delivery achieving complete tumor response with no residual disease at initial histopathology follow up in these patients. Overall, our approach potentiates PDT as a viable therapeutic option for early stage oral lesions that can be delivered in low resource settings.

## Introduction

The incidence of oral cavity cancer has increased globally in the past decade. The mortality rates are especially alarming in developing countries, such as those of South Central Asia, due to high prevalence of chewing tobacco^1,2^. In fact, almost 33% of cancers reported in the high poverty regions of Southeast Asian countries (Bangladesh, India, Pakistan and Sri Lanka) are in the oral cavity. Moreover, these regions lack resources for early detection, leaving fewer therapy options for advanced disease. Despite being only marginally effective, surgery, radiation and chemo therapies are still mostly unavailable in these low to middle income countries. There is a critical demand for an effective treatment modality that can be transparently adapted to low-resource settings.

Amongst the various targeted therapies being explored for oral cancer, Photodynamic Therapy (PDT) has demonstrated promising clinical results with significant epithelial necrosis and excellent healing after treatment^3–17^. PDT is a light based cytotoxic therapy that causes targeted damage when a photosensitizer molecule accumulated in the tumor interacts with a specific wavelength of light to generate reactive species. Relative to surgery and radiation, PDT is non-disfiguring and has minimal side effects and patients treated with PDT have not reported a loss in sensation within the oral cavity. These considerations all point to the potential benefit to patients using PDT for small and/or early stage oral lesions for which light penetration through tissue is sufficient to achieve complete tumor response without the need for surgery. Furthermore, given that photosensitizer and light delivery, do not require extensive medical infrastructure, PDT is inherently conducive for adaption into low-resource areas^18,19^.

Notwithstanding demonstrated clinical promise, particularly for early stage disease, the implementation of PDT for treatment of oral malignancies has been limited to a relatively small community of clinicians with expertise in laser medicine. This underutilization may be attributed in large part to the critical importance of accurate dosimetry in achieving good PDT outcomes^20^, combined with a lack of streamlined intraoral light delivery solutions to enable this. Dosimetry involves delivering precise and uniform light (no spatial non-uniformities) in specific regions of the mouth for prolonged time periods, often more than an hour. The total light dose delivered and the effective fluence rate on the lesion is highly dependent on the distance between the light source and target tissue. With longer illumination time for PDT, maintaining consistent light source to target tissue in conscious subjects is a challenge. In general, the subjects are sedated with physician holding the light delivery fiber during the therapy or utilizing inconvenient holders^21^. Translating this into a “low resource settings” procedure that can be performed as an “outpatient” visit will involve meticulous planning with excessive inconvenience to the subject. Devices such as reflectors and light pipes have been tailored to facilitate stable light delivery in sedated patients^21^. However, these devices are mostly metal, are not flexible/nonconformable, and are not customized to the patients’ facial and oral dimensions. Furthermore, these devices are designed for treatment of “flat” lesions such as those on the tongue but not for irregular or curved surfaces such as the lesions in the retromolar region.

The specific goal of this study was to evaluate the ergonomics (comfort and stability) and clinical utility of our 3D printed oral applicators for PDT in conscious subjects that address the challenges stated above for precise and uniform light delivery in PDT. We designed conformable, non-metal oral applicators (inspired by bite wings used in dentistry) to deliver light stably to the lesions after extensive discussion with clinicians in the USA and India. Here, the natural structure of the patients’ oral cavity, teeth and jaw provided the support and stability to hold the fiber in place, avoiding any use of posts, holders, reflectors or light pipes. To evaluate the stability and comfort of these applicators, we replaced the light delivery fiber with a similar sized endoscope to record motion of a fiducial ink marker in the anterior buccal, posterior buccal mucosa or retro molar region in the mouth. The recorded videos were analyzed for displacement in a 10-minute window and the patients were asked to rate their comfort levels. Finally, utilizing these applicators, we performed a pilot clinical study in five subjects with T1N0M0 oral cancer lesions that demonstrated no lymph node involvement. Patients were treated with PDT and demonstrated no cancerous lesions several months after therapy, as supported by immunohistochemistry. None of these cases had scarring or fibrosis post treatment. Overall, this work offers an engineered methodology to deliver light stably in the oral cavity for appropriate PDT dosimetry.

## Materials and Methods

### Fabrication of oral applicator

**Figure 1:**
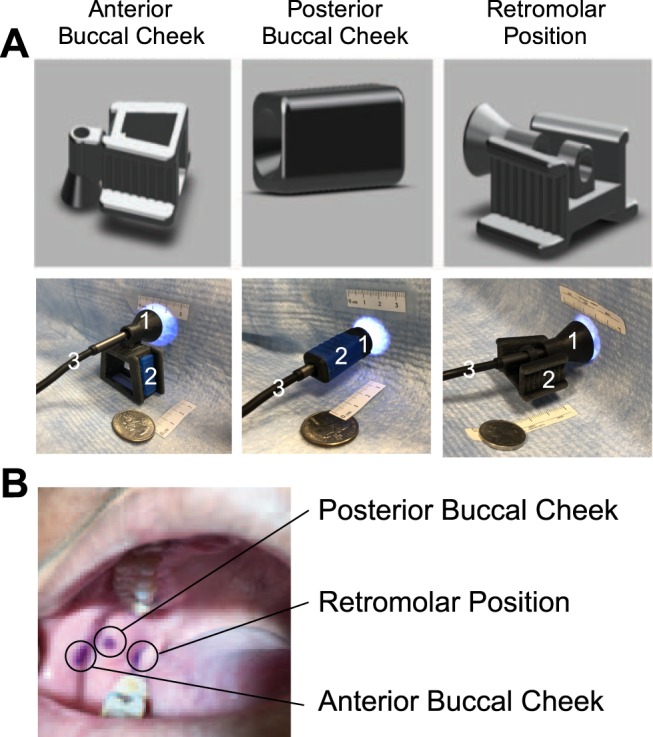
(**A**) 3D Schematics and photograph of the applicators for three different regions in the mouth Anterior buccal cheek position, posterior buccal cheek position and retromolar region. The photographs showcase the integrated unit with the applicator (1), the bite wing (2) and the endoscope (3) utilized in the ergonomics clinical study. For photodynamic therapy in patients, we replaced the endoscope with a fiber that delivered 635 light from our custom designed light source. (**B**) Photograph of the oral cavity with ink marks in three points tested in the ergonomics study.

Intraoral applicators that attach to the optical fiber were 3D printed with modular design. The modular design consisted of two parts, the applicators and the bite blocks. Appropriate applicator that delivers beam spot (with pre-calibrated dosimetry) suitable for a particular lesion size can be coupled with bite blocks that position the applicator angle for a particular lesion position. Considering the complexity of the oral cavity, we designed different applicators (Fig. 1A) for three locations, namely anterior/posterior buccal or retromolar region as shown in Fig. 1B. The oral light applicators are made of two parts: the bitewings and light delivery end. The bitewings are modeled after the standard dental clinical bite blocks for ergonomic fitting. It has additional fiber mounting feature to facilitate flexible light delivery (Fig. 1A). The three bite wings were designed in standard child, middle and adult size, similar to the sizes routinely used in dental clinics. The design of the light delivery piece is based on free space optics which allows the beam to reach its desired size of 1–2 cm in diameter. The cone shaped applicator can also help support the targeted tissue by pressing onto it. A customized fiber connector can screw into the light delivery pieces directly. We made three sizes of light delivery pieces which can deliver light to 1 cm, 1.5 cm or 2 cm in diameter respectively. A photograph of the large adult size bite wing (to deliver light to the anterior buccal cheek location) fitted with an endoscope is shown in Fig. 1A. Some of the applicators are inter-changeable or multi-purpose. i.e., the rectangular applicator designed for posterior buccal cheek position could also be used for retromolar positioning depending on the jaw and oral anatomy of the subject. The light applicator devices were designed using Autodesk Fusion 360 and are produced by a 3D printer (Object 30 pro, Stratasys). We used vero blue and vero black filament for the printing material, which gave a solid but a little elastic texture. For each set of applicators, about 20 g of printing material was used. After 3D printing, the light delivery devices were cleaned with high-pressure water. Before the clinical use and in between clinical testing, the applicators were sterilized by completely submersing them in *Cidex OPA* disinfectant solution for at least 12 minutes at 20 °C to achieve high-level disinfection. Following removal from *Cidex OPA* solution, the applicators were thoroughly rinsed with distilled water and dried before use^22^.

### Ergonomics study details

To evaluate the comfort and stability of applicators, we performed a study approved by the Massachusetts General Hospital Partners Institution Review Board on 10 subjects. The applicators for anterior buccal cheek, posterior buccal cheek or retromolar positions were covered in a hygienic disposable sleeve (Defend Air/Water Syringe Sleeves 2.5 × 10 in) along with the endoscope (USB Endoscope fitted with 5.5 mm diameter camera and 6 LEDs for illumination by 10DM, Amazon) and positioned for 10 minutes each. Subjects were asked to rate comfort and fatigue on a numerical scale of 1 to 5 where 1 was no discomfort due to the applicator and 5 was intolerable discomfort due to the applicator. The subjects were specifically asked three questions: 1. Physical discomfort: Was there any physical discomfort at any point during the 10 minutes, 2. Rate the fatigue or numbness in the mouth and 3. Repeatability: Would you be comfortable immediately repeating another 10-minute interval at the same site? The applicators were fitted with an endoscope that recorded positions of ink marks at target spots (anterior/posterior buccal and retromolar) during the testing period to evaluate stability of the applicators. To assess positioning stability, the recorded videos were processed with custom designed algorithms in MATLAB to track movement of ink marks. The sequence of positions tested was randomly chosen for every patient (Suppl. Fig. 1).

Subjects gave written and informed consent to participate in all aspects of this study in compliance with the US Federal Code of Regulations pertaining to conduct of clinical studies. This study was conducted according to good clinical practice guidelines and the declaration of Helsinki and was approved by the Institutional Review Board of Massachusetts General Hospital.

### Endoscope video and Image processing

**Figure 2:**
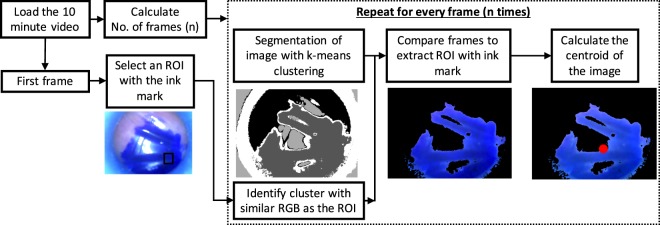
Flow chart of video and image processing method to calculate centroid of the fiducial ink mark mimicking the anterior buccal cheek, posterior buccal cheek or retromolar position imaged with a USB Endoscope fitted to the oral applicators.

To evaluate the comfort and stability of applicators (Fig. 1), we performed a study on 10 subjects where the physician placed three ink marks on the inner cheek of the subjects. The applicators for the three positions, namely the anterior buccal cheek, posterior buccal cheek and retromolar positions (Fig. 1B), were tested one after another for 10 minutes. The applicators were fitted with a semi-rigid wireless endoscope (10 DM USB Endoscope, 5.5 mm Borescope Inspection Camera with 6 LED lights, Amazon Inc #B07FPB3HG4) that recorded the movement of the ink marks during the testing period to evaluate stability of the applicators. The recorded videos were processed with custom designed algorithms in MATLAB as shown in Fig. 2. Specifically, the ink mark is isolated from the rest of the regions (applicator, healthy tissue) using k means cluster analysis for every frame in the video. The cluster region with similar Red, Green, and Blue values as the ROI of the first frame is identified and the centroid is calculated for analyzing movement in the ink mark.

### Photodynamic therapy study details

Five eligible patients with confirmed T1N0M0 oral lesions at JN Medical College in Aligarh India were recruited for the study. Informed consent was obtained from the subjects. Briefly, patients were given the photosensitizer precursor 5-ALA (DUSA Pharmaceuticals) using a well-established administration protocol of three 20 mg/kg doses dissolved in orange juice within two hour interval^11,23,24^. ALA-induced PpIX causes cutaneous photosensitivity, so avoidance of inadvertent exposure to sunlight or artificial light is recommended for 24–48 hours. Hence patients were provided with the appropriate clothing, including a full sleeve gown, to cover the skin completely. In addition, patients also received sunglasses to wear and were advised to stay indoors. The photosensitized lesions were then imaged using the smartphone-based fluorescence device, as previously published by us^19,25^ to guide light applicator placement. Two hours after ALA administration, a total light dose of 100 J/cm^2^ was delivered over approximately 35 minutes with brief breaks in light delivery every ten minutes. Post treatment, fluorescence photographs were obtained again to evaluate the photobleached area due to PDT. All clinical procedures were in compliance with local institutional guidelines at Aligarh Muslim University and received prior approval from the Indian Council on Medical Research (ICMR) and Institutional ethical committee.

## Results and Discussion

### Comfort of the applicators

**Figure 3:**
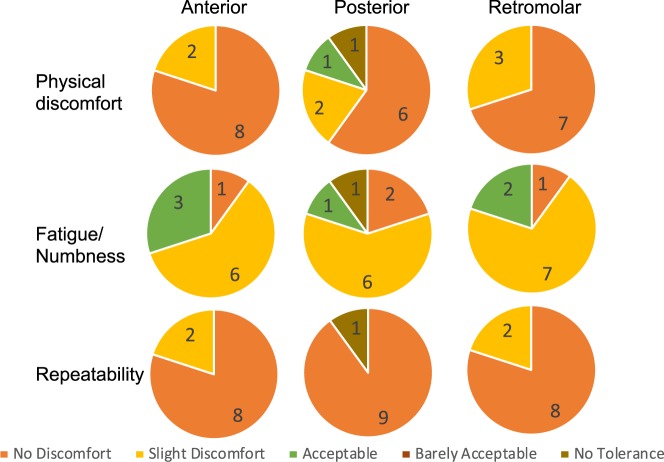
Graphical representation of the scores given by the subjects post 10 minute testing of the applicators.

The comfort levels and the numbness in the mouth of the subjects for the anterior buccal cheek, posterior buccal cheek and retromolar position is shown in Fig. 3. The subjects rated the applicators to be comfortable overall. The average scale of discomfort (1 no discomfort and 5 was no tolerance) was 1.2 +/− 0.42, 1.8 +/− 1.32, 1.3 +/− 0.48 for the anterior buccal cheek, posterior buccal cheek and retromolar position respectively. Only one subject reported “no tolerance” to the applicator in the posterior buccal cheek position. The subjects had bearable fatigue and numbness in the area post study. The average fatigue and numbness values (1 being no fatigue to 5 being extreme fatigue) were 2.2 +/− 0.63, 2.2 +/− 1.1, 2.1 +/− 0.57 for the anterior buccal cheek, posterior buccal cheek and retromolar position respectively. The same subject with no tolerance to the applicator in the posterior buccal cheek position had extreme fatigue for similar position as expected. The subjects readily agreed to repeat the procedure and conveyed minimal discomfort to do so. Specifically, the average repeatability score (1 no discomfort in repeating the procedure and 5 was no tolerance to the procedure) was 1.2 +/− 0.42, 1.4 +/− 1.26 and 1.2 +/− 0.42 for the anterior buccal cheek, posterior buccal cheek and retromolar position respectively. There were no subjects in the “No tolerance group” for the anterior buccal cheek position and the retro molar position applicators. The retromolar and posterior positions were the most comfortable applicators.

### Stability of the applicators

**Figure 4:**
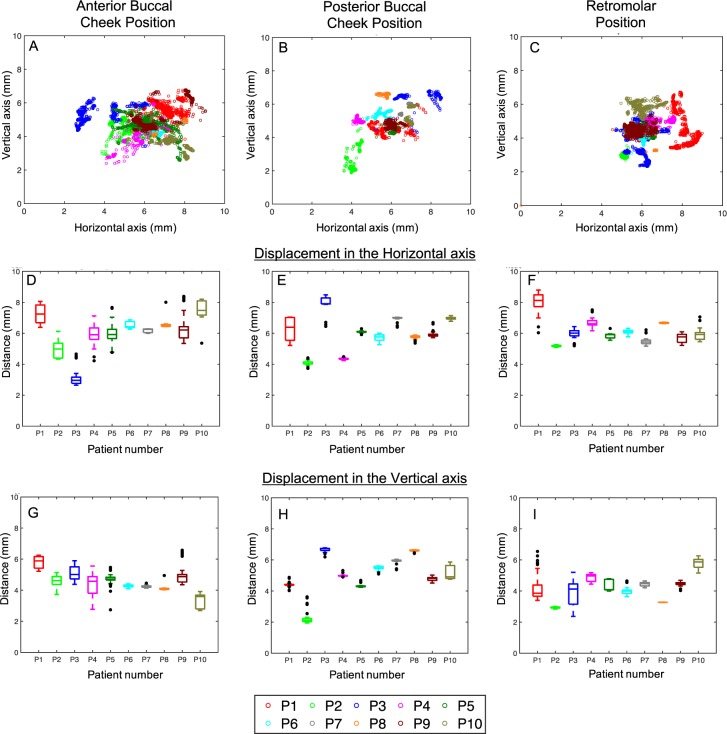
(**A**–**C**) Location of the fiducial ink mark centroid obtained from the post-processing of all the video frames from the 10 subjects. The horizontal (**D**–**F**) and vertical displacements (**G**–**I**) for the anterior buccal cheek position (left panel), posterior buccal cheek position (center panel) and the retromolar position (right panel) for the 10 subjects displayed as boxplots with mean of the centroid location in the 10 minute video, 25–75% percentile bars and outliers (black circles) that occur during any swallowing or other oral cavity motion.

The centroid of the fiducial ink mark from the customized segmentation algorithms for all the frames in the videos of the ten subjects is plotted in Fig. 4. A movie displaying the overlay of the video frames and the centroid (red circle) calculated from the custom image processing algorithm is provided in the Supplementary data. The 10 minute video obtained post processing of the centroid is downsampled to 40 seconds for viewing purposes. As seen in the video and the graphs in Fig. 4, the centroid of the ink mark has remained within the range of 1.65 ± 1.08 mm, 0.84 ± 0.68 mm, 1.45 ± 0.99 mm in the horizontal direction (Fig. 4D–F) and within the range of 1.3 ± 0.97, 0.6 ± 0.47, 1.2 ± 1 mm in the vertical direction collectively for all subjects in the anterior, posterior and retro molar positions respectively. The mean standard deviation in the centroid position for all the subjects was 0.44 ± 0.27 mm, 0.23 ± 0.23 mm, 0.26 ± 0.17 mm in the horizontal direction and 0.33 ± 0.23, 0.14 ± 0.12 mm, 0.28 ± 0.26 mm in the vertical direction (Fig. 4G–I) for the anterior, posterior and retro molar positions respectively. These results showcase that the applicator is the most stable for the posterior buccal check position, i.e., less deviation in the horizontal and vertical motion. We attribute this to the smaller size of the applicator in this position than in other two positions (Fig. 1). Though the patients had similar discomfort levels for all three applicators, the larger width of the anterior buccal cheek and retro molar position applicators could be the reason for the extra motion of the fiducial marker in the video. The subject could have either “swallowed saliva” or adjusted their jaws to accommodate the larger width of the anterior buccal cheek and retro molar position applicators. The “swallowing” action causes the cheek muscles to move horizontally and indeed we can note that the horizontal motion for the larger applicators is almost twice higher than the posterior buccal cheek applicator motion. Overall, the applicators were comfortable and had minimum motion allowing stable light delivery for treatment.

### Light delivery was beyond the tumor margins leading to excellent treatment outcome

**Figure 5:**
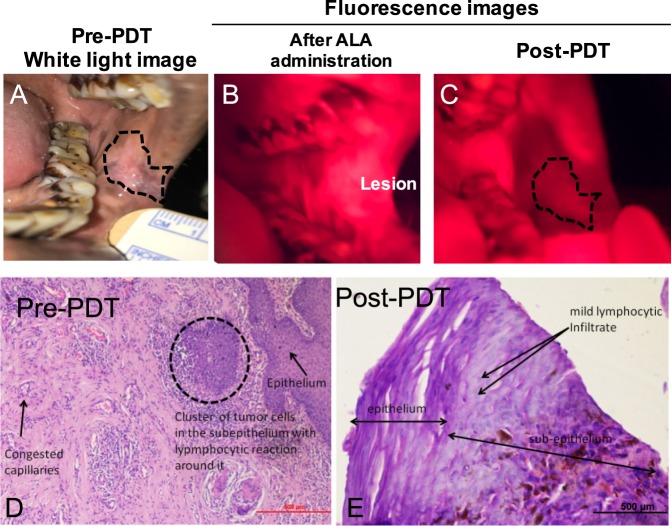
(**A**) Pretreatment regular white light photograph of the lesion (outlined in black dotted line). (**B**,**C**)Fluorescence image of the PpIX accumulation (pre light irradiation) and photobleaching in the lesion region post irradiation. (**D**–**E**) Histology images of the biopsy taken before and after the treatment showcase absence of malignant cells post therapy.

Photosensitizer is a theranostic agent. The change in photosensitizer fluorescence before and after light irradiation (Photobleaching due to PDT light irradiation) has been correlated to treatment response in several applications^20,26–28^. Aminolevulinic acid converts to photosensitizer protoporphyrin (PpIX) is malignant tissue compared to healthy tissue. Utilizing this concept, we evaluated if light delivery was beyond the tumor margins by comparing pre-treatment white light and fluorescence images to Post-PDT fluorescence images. Table 1 shows the details of the patients that were treated with our custom-built, low cost light source (published previously)^18,19^ and light applicators described in this manuscript. Representative data from a 62 y/o female patient with squamous cell carcinoma in the left posterior buccal cheek position is shown in Fig. 5. The fluorescence images of the oral cavity pre-PDT (Fig. 5B) shows us higher accumulation of the protoporphyrin (PpIX), specifically in the lesion area and subsequent photobleaching (darkening of the region) of PpIX post light irradiation (Fig. 5C). The original lesion area is overlaid on the post-PDT image to demonstrate the motion in the fiducial ink mark. In all the subjects, the photobleached area (calculated from the fluorescence images) was greater than the lesion area by ~3.7 ± 1.5 cm^2^. Furthermore, biopsies from post-treatment follow-up of the patient showed no signs residual or recurrent cancer and visually excellent healing of the oral mucosa was observed. Importantly, there was no fibrosis observed one-year post treatment. This was noted consistently with all these patients having early cancer (shallow lesion size) and is in agreement with previously reported observations^4,8,9,16^ with expensive lasers and light delivery systems.

**Table 1:** Details of the patient age, gender, location and area of the lesion treated with low cost, battery powered light source and custom designed applicators.

Patient Age/Gender	Tumor location on Buccal Mucosa (BM)/area (cm^2^)	Photobleached area > lesion area (cm^2^)
62 years/F	Left posterior BM/0.871	Yes, 3.94
28 years/M	Left posterior BM/1.01	Yes, 3.80
40 years/M	Right anterior BM/1.62	Yes, 3.93
53 years/M	Left anterior BM/1.02	Yes, 5.26
45 years/M	Left anterior BM/1.55	Yes, 7.69

Carcinoma of the buccal mucosa is the most common oral cavity cancer in India, mainly caused by the widespread use of the gutkha or betel quid chewing (with or without tobacco)^29^. The fiber-coupled applicator system proposed in this study was designed for light delivery to the buccal cheek and retromolar lesion sites as these sites are the common places where people tend to retain the gutkha or the betel quid for extended time periods leading to high propensity for cancer occurence. To deploy the applicators for clinical use, we focused on these sites; however, in future, we plan to develop diverse applicator designs for treating additional lesion sites like tongue or the floor of the mouth. Furthermore, in our current study, we focused on lesions that are 1 cm or less in diameter (Table 1) to enable treatment of the whole lesion in “one sitting.” Larger lesions could be treated and we are currently pursuing these directions by either redesigning the applicators to irradiate larger lesion area in one-sitting or perform multiple treatments of overlapping smaller areas to cover the complete lesion. The first option of redesigning the applicators to deliver light to larger lesions will either increase the size of the applicators or potentially could reduce the fluence (thereby causing an increase in irradiation time). Currently we are working on improving the output fluence of the light source that can be used for larger lesions. The second option to perform sequential treatments of smaller areas will require the applicator to be moved or adjusted to irradiate various locations within the lesion. Though this could be done under image-guidance (such as ultrasound or fluorescence) as the various applicator positions in the mouth have to be accurately designed to avoid under- or over-treating regions^20^. Our future work will involve addressing these issues while providing treatments with low-cost systems.

## Conclusions

Here we demonstrated the utility of a system involving modular, non-metal, flexible, and quasi-customizable, applicators and associated mouth props to deliver light to lesions for PDT treatment in conscious subjects. While we used a set of applicators with pre-determined sizes that were comfortable for various subjects, mouth and jaw dimensions and genders, it is reasonable to envision extension of this approach to customized patient treatment. Specifically, personalized applicators can be rapidly printed at the time of procedure due to advances in image-based 3D printing and the increasing availability of low-cost, high-quality 3D printers in clinical settings. The personalization of the applicators based on patients’ facial and oral cavity dimensions is a quicker and comfortable procedure than utilizing uncomfortable and non-flexible metal light delivery systems. The ergonomic design of 3D printable light applicators has significant practical benefit in enabling longer irradiation duration and improved accuracy of light delivery necessary for curative PDT. With costs of healthcare and cancer incidences increasing worldwide, particularly in developing countries, we report an affordable methodology for delivering light stably and ergonomically in the oral cavity which can be used in conjunction with a low-cost, portable, battery-powered fiber-coupled LED based light source^25^. The potential for impact is high, especially in developing countries where PDT may fill the unmet need for a low-cost and non-scarring intervention that can be deployed at resource-limited primary care sites to patients whose disease would otherwise advance to an unmanageable stage. Although evaluated here in a setting where low-cost and portability of the light source are important, the 3D printed intraoral applicators described here provide customizability and versatility to couple to any standard existing fiber-based medical laser sources to enable intraoral PDT even in the best-equipped clinical settings. Our preliminary studies on patients with early-stage oral lesions suggest that this overall approach is comfortable, user-friendly, and effective with potential for individualized treatment. Future studies will focus on clinical evaluation of the treatment of larger lesions and personalizing the dosimetry based on photosensitizer uptake.

## Supplementary information

Supplementary Video

Supplementary Data
