# Supplementary material for: Platform for ergonomic intraoral photodynamic therapy using low-cost, modular 3D-printed components: Design, comfort and clinical evaluation
Source: Sci Rep. 2019 Nov 1;9:15830. doi: 10.1038/s41598-019-51859-6 (PMC6825190; doi:10.1038/s41598-019-51859-6)
Supplement: Supplementary file 2 — Supplementary Data [file 41598_2019_51859_MOESM2_ESM.docx]

**Platform for ergonomic intraoral photodynamic therapy using low-cost, modular 3D-printed components:**

**Design, comfort and clinical evaluation**

Srivalleesha Mallidi^1, #, ##^, Amjad Khan^1,#^, Hui Liu^2,^ , Liam Daly^3^, Grant Rudd^3^, Paola Leon^3^,

Shakir Khan^4^, Bilal Hussain^4^, Syed A. Hasan^5^, Shahid A. Siddique^4^, Kafil Akhtar^6^,

Meredith August^7^, Maria Troulis^7^, Filip Cuckov^3^, Jonathan Celli^2^ and Tayyaba Hasan^1,*^

^1^ Wellman Center for Photomedicine, Massachusetts General Hospital, Harvard Medical School, Boston, Massachusetts, USA.

^2^ Department of Physics, University of Massachusetts at Boston, Boston, Massachusetts, USA.

^3^ Department of Engineering, University of Massachusetts at Boston, Boston, Massachusetts, USA

^4^ Department of Radiotherapy, Jawaharlal Nehru Medical College, Aligarh Muslim University, Aligarh, India

^5^ Department of Oto-Rhino-Laryngology, Jawaharlal Nehru Medical College, Aligarh Muslim University, Aligarh, India

^6^ Department of Pathology, Jawaharlal Nehru Medical College, Aligarh Muslim University, Aligarh, India

^7^ Department of Oral and Maxillofacial Surgery, Massachusetts General Hospital, Boston MA, USA

*To whom correspondence should be addressed: [thasan@partners.org](mailto:thasan@partners.org)

^#^Equal contribution

## Current affiliation: Department of Biomedical Engineering, Tufts University Medford Massachusetts, USA

**Supplementary information**

**Figure 1**

| **** |
| --- |
| **Fig. S1:** Graph depicting the order of evaluating the application in the subjects of the ergonomics clinical study**.** |

**Movie:** Suppl. Movie 1 480px.mov
